# Learning climate positively influences residents’ work-related well-being

**DOI:** 10.1007/s10459-018-9868-4

**Published:** 2018-12-05

**Authors:** Lenny S. S. Lases, Onyebuchi A. Arah, Olivier R. C. Busch, Maas Jan Heineman, Kiki M. J. M. H. Lombarts

**Affiliations:** 1 Professional Performance Research Group, Department of Medical Psychology, Academic Medical Center, University of Amsterdam, Amsterdam, The Netherlands; 2 Department of Surgery, Isala, Zwolle, The Netherlands; 3 Department of Epidemiology, Fielding School of Public Health, University of California Los Angeles (UCLA), Los Angeles, CA USA; 4 Department of Surgery, Academic Medical Center, University of Amsterdam, Amsterdam, The Netherlands; 5 Academic Medical Center, University of Amsterdam, Amsterdam, The Netherlands

**Keywords:** Residency training, Learning climate, Residents’ well-being, Work-engagement, Job satisfaction

## Abstract

An optimal learning climate is crucial for the quality of residency training and may also improve residents’ well-being and empathy. We investigated the associations of learning climate with residents’ work-related well-being. A multicenter questionnaire study was performed among 271 surgery and gynaecology residents in 21 training programs from September 2012 to February 2013. Residents were asked to complete work-related well-being measurements: work engagement (Utrecht Work Engagement Scale), job and specialty satisfaction (measures from Physician Worklife Study), and physician empathy (Jefferson Scale of Physician Empathy). The Dutch Residency Educational Climate Test was used to evaluate learning climate. Multivariate adjusted linear regression analyses were used to estimate associations of learning climate with work-related well-being measures. Well-being measures were completed by 144 residents (53.1%). Learning climate was evaluated by 193 residents, yielding 9.2 evaluations per training program on average. Overall learning climate score was positively associated with work engagement [regression coefficient *b* = 0.58; 95% confidence interval (CI) 0.18–0.98; *p* = 0.004] and job satisfaction (*b* = 0.80; 95% CI 0.48–1.13; *p* < 0.001). No associations were found between learning climate and empathy and specialty satisfaction. Residents’ work engagement and job satisfaction are positively related to the learning climate and may be further enhanced by improved learning climates of training programs.

## Introduction

Physicians’ well-being is presumed to be a quality indicator for health care systems (Wallace et al. 2009). This topic is receiving worldwide attention as research suggests that physicians are experiencing high work-related pressure. This experienced high pressure is detrimental to the experienced well-being, which could have negative individual and professional consequences (Wallace et al. 2009; Prins et al. 2009; West et al. 2006). Work engagement and work satisfaction are important aspects of work-related well-being and are found to be related to professional performance and quality of patient care. Work engagement could be defined as a positive, fulfilling, work-related state of mind and the opposite of burn-out (Schaufeli and Bakker 2003; Schaufeli et al. 2002). Engaged physicians report fewer medical errors, perform better and show more adequate safety behaviors (Prins et al. 2009; Scheepers et al. 2015b; Biddison et al. 2016; Mache et al. 2013). Likewise, physicians’ work satisfaction is associated with better patient care delivery and more satisfied patients (Williams et al. 2007; Williams and Skinner 2003). In general, a lack of work-related well-being or experiencing distress could lead to a decrease in physicians’ empathy (Ahrweiler et al. 2014; Neumann et al. 2007). This is unfortunate because decreased empathy will inhibit physicians’ ability to understand, communicate, and respond to patients’ perspectives and experiences, thus reducing patient satisfaction, compliance and safety (Kim et al. 2004; West et al. 2006; Neumann et al. 2012; Mercer and Reynolds 2002; Hojat 2007; Hojat et al. 2002b).

It is essential to understand which factors influence physicians’ well-being. Besides working irregular hours and a lack of autonomy, workload is viewed as one of the major aspects negatively influencing work-related well-being (Wallace and Lemaire 2007; Prins et al. 2007a; Wallace et al. 2009). Social support, receiving performance feedback, having a positive impact on patients’ lives and successful patient outcomes, however, are factors found to be positively related to physicians’ well-being (Wallace and Lemaire 2007; Shapiro and Galowitz 2016; Prins et al. 2007b).

In addition, a less healthy learning climate was recently shown to be associated with burnout among residents, lowered quality of life in orthopedic trainees, and medication errors among nurses (van Vendeloo et al. 2014; Llera and Durante 2014; Chang and Mark 2011). Learning climate in residency training contains the formal and informal aspects of education (Roff and McAleer 2001). However, there is little research linking learning climate and its distinct domains to a broader set of measures of work-related well-being and to empathy in residency training. This study aims to fill this void.

The learning climate is known to be crucial for optimizing training outcomes, and viewed as an important quality indicator of postgraduate medical education programs (Weiss et al. 2012, 2013; WFME 2003). It can be measured with well-researched and reliable tools. We used the Dutch Residency Educational Climate Test (D-RECT), consisting of nine separate learning climate domains: educational atmosphere, teamwork, role of specialty tutor, coaching and assessment, formal education, resident peer collaboration, work adaptation to residents’ competence, accessibility of supervisors, and patient sign out (Boor et al. 2011; Silkens et al. 2016). Besides the importance for the quality of residency training, it is key to investigate the impact of the learning climate on residents’ work-related well-being and empathy. Especially, given the fact that physicians’ work-related well-being and empathy are crucial for the quality of patient care (Wallace et al. 2009; Prins et al. 2009; Williams et al. 2007; Llera and Durante 2014; van Vendeloo et al. 2014; Scheepers et al. 2015b; Biddison et al. 2016; Mache et al. 2013; Kim et al. 2004; West et al. 2006).

We hypothesize that a positive and supportive learning climate improves residents’ work engagement and their feeling of work satisfaction. Additionally, we postulate that a positive and supportive learning climate facilitates the development of residents’ ability of understanding others’ perspectives and communicate this understanding and is therefore positively associated with residents’ empathy. Thus, in this study, we aim to investigate the association of learning climate with (1) residents’ work engagement, (2) job and specialty satisfaction and (3) empathy.

## Methods

### Study population and setting

We performed a cross-sectional, multicenter questionnaire study and invited 271 surgery and gynaecology residents, in 21 residency training programs, from two academic and 14 non-academic medical centers in the Netherlands. The residents were invited to participate in a web-based survey by email between September 2012 and February 2013. For most residents the work-related well-being measures and the learning climate measure were administered at the same time. However, in some training programs, the measures were administered in a different order depending on logistic reasons and choice of the program director.

### Measures

#### *Work*-*related well*-*being* (outcomes): work engagement, job and specialty satisfaction and empathy

To measure work engagement of the residents we used the short version of the Utrecht Work Engagement Scale (UWES-9). This reliable and widely used scale consists of nine items on three domains (vigor, absorption and dedication). The items are measured on a 7-point scale (0 = never, 1 = almost never, 2 = rarely, 3 = sometimes, 4 = often, 5 = very often, 6 = always/daily) (Schaufeli and Bakker 2003; Sepällä et al. 2009; Scheepers et al. 2015a).

The global job and specialty satisfaction measures from the Physician Worklife Study were used to measure work satisfaction of the residents (Williams et al. 1999; Konrad et al. 1999). The two global measures evaluate job satisfaction and specialty satisfaction with five and three items respectively. Each item could be rated on a 5-point Likert scale (see “Appendix” document for the items of the global job satisfaction and global specialty satisfaction measures). The scale ranges from 1 (strongly disagree) to 5 (strongly agree). Translation of these measures into Dutch was carried out by three of the authors (SSL, MJMHL, OAA) using the forward-back-translation procedure (Brislin 1970).

We used the Jefferson Scale of Physician Empathy (JSPE) to evaluate residents’ empathy (Hojat et al. 2002a, b; Glaser et al. 2007). This instrument contains 20 items, which can be rated on a 7-point Likert scale that ranges from 1 (strongly disagree) to 7 (strongly agree). This instrument was also translated into Dutch following the appropriate forward-back-translation procedure (Brislin 1970).

#### *Learning climate* (predictor variable)

We used the Dutch Residency Educational Climate Test (D-RECT) to evaluate the learning climate of each residency training program (Boor et al. 2011; Silkens et al. 2016). The D-RECT is the instrument most used for measuring learning climate in residency training in the Netherlands, and other health care systems (Iblher et al. 2015; Bennett et al. 2014; Pinnock et al. 2013). Both the original D-RECT and the revised D-RECT were found to be reliable and provide valid results (Silkens et al. 2016; Boor et al. 2011). We used the latter updated structure of the D-RECT consisting 9 learning climate domains and 35 items. Each item could be rated on a 5-point Likert scale, ranging from 1 (totally disagree) to 5 (totally agree). Three resident evaluations are needed to reliably rate the overall learning climate but eight resident evaluations are needed for the separate climate domains: (1) educational atmosphere, (2) teamwork, (3) role of specialty tutor, (4) coaching and assessment, (5) formal education, (6) resident peer collaboration, (7) work adaptation to residents’ competence, (8) accessibility of supervisors, and (9) patient sign out. In this study, the individual residents’ evaluations on learning climate were aggregated to program level to represent the overall learning climate score and domain scores of each residency training program.

### Data analyses

To describe the characteristics of the setting and participating residents we first calculated the descriptive statistics. Next, we calculated the mean and median scores of the four outcome measures residents’ work engagement, empathy, job and specialty satisfaction as well as the mean and median scores of the overall learning climate and the nine specific learning climate domains.

For relating the predictor variable, namely learning climate, to the four outcome variables we performed multivariate adjusted linear regression analysis using generalized estimating equations (GEEs). We performed separate analyses for the overall learning climate and the nine learning climate domains with the outcome variables. Using GEE allowed us to account for cross-clustering or nesting of the evaluations (Gelman and Hill 2007). We accounted for cross-clustering of residents’ perceptions of the learning climate within residency training programs and hospitals. Additionally, we adjusted for gender and year of residency by treating them as covariates in the analyses. Our results were reported as regression coefficients with 95% confidence intervals. We used the false discovery rate (FDR) to adjust the *p* values from the multiple testing to reduce our chances of accepting false positive results (Benjamini and Hochberg 1995; van der Leeuw et al. 2013). Statistical analyses were performed with IBM Statistics SPSS 20.0 and SAS 9.4 (SAS Inc., Cary, NC).

### Ethical approval

The institutional ethical review board of the Academic Medical Center of the University of Amsterdam was consulted and waived ethical approval. All precautions were taken to protect anonymity and confidentiality of the study participants.

## Results

The work-related well-being measurements were completed by 144 (53.1%) residents. The learning climate of 21 residency training programs was evaluated by 193 residents with a mean of 9.2 (range 4–20) residents’ evaluations per training program. As described in the methods, we aggregated these residents’ evaluations on learning climate to program level to represent the overall learning climate score and domain scores of each residency training program. The characteristics of the study setting and participants are described in Table 1. Table 2 shows the residents’ scores on work engagement, job and specialty satisfaction, empathy and the learning climate scores including the different domains on training program level.

**Table 1:** Characteristics of study setting and participants

	Variable	N	%
Setting	Teaching hospitals		
	Academic	2	
	Non-academic	14	
	Residency training programs	21	
	Residents evaluations of learning climate per training program, mean (min–max)	9.2 (4–20)	
Participants	Specialty		
	Surgery	111	77.1
	Gynaecology	33	22.9
	Gender		
	Male	74	51.4
	Female	65	45.1
	Missing	5	3.5
	Year of residency		
	0–3 years	92	63.9
	4–6 years	47	32.6
	Missing	5	3.5

**Table 2:** Residents’ engagement, empathy, job and specialty satisfaction scores and the overall and domain scores of the learning climate on training program level

Outcome variable	Measurement (scale)	N	Mean (SD)	Median (IQR)
Engagement	UWES (0–6)	142	4.44 (0.74)	4.56 (4.00–5.00)
Job satisfaction	(1–5)	142	4.12 (0.62)	4.20 (3.80–4.60)
Specialty satisfaction	(1–5)	142	4.00 (0.74)	4.00 (3.33–4.67)
Empathy	JSPE (20–140)	139	111.78 (12.44)	111 (105–121)
Learning climate	DRECT (1–5)			
Overall score		21	3.78 (0.23)	3.80 (3.62–3.93)
Educational atmosphere		21	3.81 (0.35)	3.76 (3.53–4.09)
Teamwork		21	3.91 (0.37)	3.82 (3.63–4.18)
Role of specialty tutor		21	4.00 (0.26)	4.00 (3.77–4.18)
Coaching and assessment		21	3.18 (0.24)	3.17 (3.01–3.42)
Formal education		21	3.59 (0.35)	3.54 (3.34–3.83)
Resident peer collaboration		21	4.37 (0.25)	4.38 (4.21–4.56)
Work adapted to competence		21	3.75 (0.32)	3.67 (3.50–4.04)
Accessibility supervisors		21	4.25 (0.27)	4.23 (4.11–4.40)
Patient sign-out		21	3.56 (0.43)	3.45 (3.38–3.84)

The results of the multivariate adjusted linear regression analysis to evaluate the associations of learning climate with work engagement, job and specialty satisfaction, and empathy are shown in Table 3. The overall learning climate was positively associated with work engagement [regression coefficient *b* = 0.58; 95% confidence interval (CI) 0.18–0.98; *p* = 0.004] and with job satisfaction (*b* = 0.80; 95% CI 0.48–1.13; *p* < 0.001). We found no association of the overall learning climate with specialty satisfaction or empathy.

**Table 3:** Regression coefficients and 95% confidence intervals for the associations of departments’ overall learning climate with residents’ work engagement, job and specialty satisfaction, and empathy

Outcome variable	Regression coefficient	Standard error	95% CI	*p* value	FDR adjusted *p* value
Engagement	0.58	0.20	0.18 to 0.98	0.004	0.016
Job satisfaction	0.80	0.16	0.48 to 1.13	< 0.001	0.005
Specialty satisfaction	0.16	0.20	− 0.24 to 0.55	0.438	0.545
Empathy	3.68	3.68	− 3.53 to 10.89	0.317	0.423

*FDR* false discovery rate

Focusing on the learning climate domains, Table 4 shows the associations between the different learning climate domains and work engagement, job and specialty satisfaction and empathy. Work engagement was found to be positively related to the learning climate domains educational atmosphere (*b* = 0.34; 95% CI 0.11–0.58; *p* = 0.004) and formal education (*b* = 0.37; 95% CI 0.14–0.60; *p* = 0.001). Educational atmosphere was also positively related to job satisfaction (*b* = 0.45; 95% CI 0.16–0.74; *p* = 0.003). Additionally, the results show positive associations for the following learning climate domains with job satisfaction: teamwork (*b* = 0.48; 95% CI 0.29–0.66; *p* < 0.001), role of specialty tutor (*b* = 0.61; 95% CI 0.25–0.97; *p* = 0.001), resident peer collaboration (*b* = 0.62; CI 0.38–0.85; *p* < 0.001), work adapted to competence (*b* = 0.63; 95% CI 0.44–0.83; *p* < 0.001) and accessibility supervisors (*b* = 0.48; 95% CI 0.20–0.76; *p* = 0.001). We found no associations between the different learning climate domains and specialty satisfaction or empathy. Controlling the false discovery rate in our multiple testing left all but one of these findings intact (see role of specialty tutor and engagement in Table 4).

**Table 4:** Regression coefficients and 95% confidence intervals for the associations of the different domains of learning climate with residents’ engagement, job and specialty satisfaction and empathy

Outcome variable	Regression coefficient	Standard error	95% CI	*p* value	FDR adjusted *p* value
Educational atmosphere					
Engagement	0.34	0.12	0.11 to 0.58	0.004	0.016
Job satisfaction	0.45	0.15	0.16 to 0.74	0.003	0.015
Specialty satisfaction	0.05	0.15	− 0.24 to 0.34	0.742	0.822
Empathy	1.79	2.40	− 2.91 to 6.49	0.456	0.553
Teamwork					
Engagement	0.19	0.12	− 0.04 to 0.42	0.097	0.233
Job satisfaction	0.48	0.09	0.29 to 0.66	< 0.001	0.005
Specialty satisfaction	0.07	0.12	− 0.16 to 0.31	0.532	0.626
Empathy	3.25	2.61	− 1.87 to 8.37	0.213	0.381
Role of specialty tutor					
Engagement	0.38	0.18	0.02 to 0.74	0.037	0.135
Job satisfaction	0.61	0.18	0.25 to 0.97	0.001	0.006
Specialty satisfaction	0.19	0.16	− 0.12 to 0.50	0.238	0.381
Empathy	− 0.47	3.04	− 6.43 to 5.48	0.876	0.899
Coaching and assessment					
Engagement	0.54	0.28	− 0.01 to 1.09	0.054	0.180
Job satisfaction	0.37	0.22	− 0.07 to 0.80	0.099	0.233
Specialty satisfaction	0.19	0.18	− 0.16 to 0.54	0.281	0.400
Empathy	6.65	3.90	− 0.99 to 14.29	0.088	0.233
Formal education					
Engagement	0.37	0.12	0.14 to 0.60	0.001	0.006
Job satisfaction	0.18	0.17	− 0.14 to 0.50	0.277	0.400
Specialty satisfaction	− 0.15	0.13	− 0.40 to 0.10	0.236	0.381
Empathy	− 0.76	3.26	− 7.16 to 5.63	0.815	0.881
Resident peer collaboration					
Engagement	0.25	0.21	− 0.17 to 0.66	0.248	0.382
Job satisfaction	0.62	0.12	0.38 to 0.85	< 0.001	0.005
Specialty satisfaction	0.15	0.15	− 0.13 to 0.44	0.290	0.400
Empathy	0.56	3.37	− 6.04 to 7.16	0.868	0.899
Work adapted to competence					
Engagement	0.19	0.15	− 0.11 to 0.48	0.210	0.381
Job satisfaction	0.63	0.10	0.44 to 0.83	< 0.001	0.005
Specialty satisfaction	0.22	0.18	− 0.14 to 0.57	0.231	0.381
Empathy	1.29	2.49	− 3.59 to 6.16	0.605	0.691
Accessibility supervisors					
Engagement	0.29	0.17	− 0.04 to 0.62	0.083	0.233
Job satisfaction	0.48	0.14	0.20 to 0.76	0.001	0.006
Specialty satisfaction	0.20	0.21	− 0.22 to 0.62	0.343	0.443
Empathy	4.51	3.73	− 2.80 to 11.83	0.227	0.381
Patient sign-out					
Engagement	0.13	0.11	− 0.08 to 0.34	0.222	0.381
Job satisfaction	0.21	0.11	− 0.01 to 0.42	0.062	0.191
Specialty satisfaction	0.01	0.11	− 0.20 to 0.22	0.929	0.929
Empathy	2.49	1.88	− 1.20 to 6.18	0.186	0.381

*FDR* false discovery rate

## Discussion

### Main findings and explanations

This study provides convincing empirical evidence on the importance of the residents’ learning climate for their work-related well-being. More specifically, we found that a more positively experienced learning climate was associated with higher residents’ work engagement and job satisfaction scores. Learning climate was neither related to residents’ specialty satisfaction nor to their levels of empathy.

The reported positive association of the overall learning climate with residents’ work engagement is in line with previous empirical studies on (the lack of) well-being, reporting that burn-out, the counterpart of work engagement, was *negatively* associated with residents’ experienced learning climate (van Vendeloo et al. 2014; Llera and Durante 2014). Theoretically, these findings can be understood within the much referenced job demands and resources (JD-R) model, which focuses on both positive and negative predictors of work-related well-being (Bakker and Demerouti 2007; Bakker et al. 2005; Schaufeli and Bakker 2004). In this JD-R model, that originated in the occupational health psychology literature, a positive predictor is referred to as a job resource, and a negative predictor as a job demand. A job resource refers to a physical, social or organizational aspect of the job that reduces job demands, is functional in achieving work goals, or stimulates personal growth, learning and development (Bakker and Demerouti 2007; Hakanen et al. 2008). Typical for a job resource is its protective potential for burnout and its stimulating potential for work engagement. Within this job demands and resources model, learning climate might be considered a job resource for residents, or more indirectly, may at least be instrumental in facilitating the uptake of other well-established job resources (Xanthopoulou et al. 2009; Hakanen et al. 2008; Schaufeli and Bakker 2004). More specifically, we assume that a positive and supportive learning climate facilitates for example in giving constructive feedback, coaching and creating educational moments. Previous research has identified these aspects as impactful job resources (Bakker and Demerouti 2007; Schaufeli and Bakker 2004).

When considering the separate learning climate domains, the positive work-related well-being construct, work engagement, seemed to be specifically related to two domains in particular, namely ‘educational atmosphere’ and ‘formal education’. A positive work atmosphere, constructive communication with faculty and structured, fitting, informative education are aspects likely to positively influence the enthusiastic, positive, fulfilling work-related state of mind (work engagement) of the residents (Hakanen et al. 2008; Xanthopoulou et al. 2009). This could explain the finding that these learning climate domains (‘educational atmosphere’ and ‘formal education’) were found to be positively associated with work engagement.

As hypothesized, we also found a positive association of the overall learning climate with job satisfaction. When looking at the learning climate domains, six out of the nine domains contributed to residents’ job satisfaction (‘educational atmosphere’, ‘teamwork’, role of specialty tutor’, ‘resident peer collaboration’, ‘work adapted to competence’ and ‘accessibility supervisors’). When re-organizing the nine learning climate domains in three higher order facets, we may distinguish the affective, the cognitive and the instrumental facet (Silkens et al. 2016; Ostroff 1993). The three climate domains making up the affective facet were found to be positively associated with job satisfaction: ‘educational atmosphere’, ‘teamwork’, and ‘resident peer collaboration’. We expect that these domains in the affective facet are rated on a more emotional basis and the positive association with the feeling of job satisfaction is therefore understandable. This is in line with research that found correlations between the affective facet of climate and satisfaction (Carr et al. 2003). On the other hand, the remaining climate domains equally divided over the cognitive and instrumental facets were not all pointing into the same positive direction of residents’ higher job satisfaction. To get a deeper understanding of what defines residents’ satisfaction with their jobs, each domain should be explored in more detail separately. For example, it may almost seem self-evident that ‘work adapted to the resident’s competence level’ and ‘accessibility of supervisors’ are positively related to job satisfaction. However, more work needs to be done to understand why ‘patient sign outs’, ‘formal education’ and residents’ ‘coaching and assessment’ seem not to have impact on residents’ satisfaction with their job.

An additional important factor that could contribute to the association of learning climate with work engagement and job satisfaction is the so-called ‘hidden curriculum’. The hidden curriculum could be defined as ‘a set of influences that function at the level of organisational structure and culture’ (Hafferty 1998), and consists of rituals, assumptions and what is implicitly taught (Mahood 2011; Gofton and Regehr 2006; Mossop et al. 2013). It has been shown that well-being experiences and professionalism of learners is influenced by the hidden curriculum (Billings et al. 2011; Rogers et al. 2012). We believe that, in residency training, and especially when talking about the learning climate, this hidden curriculum plays a substantial role and has an important impact on residents’ work engagement and job satisfaction.

We did not find an association of learning climate with residents’ specialty satisfaction or empathy. Although, learning climate is positively associated with job satisfaction, residents’ specialty satisfaction seems to be determined by other factors than the current learning climate. Specialty choice was made before entering the residency training program, and this study suggests that specialty satisfaction could be viewed as (somewhat) independent from residents’ current job satisfaction.

Lastly, this study could not confirm the association between positive learning climate and residents’ empathy levels. The debate on whether or not physicians’ empathy can be developed, and its potential facilitating factors is still ongoing (Ahrweiler et al. 2014; Neumann et al. 2011; Pedersen 2010). Although some argue that empathy is an inborn trait, multiple studies have indicated that empathy may be developed by targeted education (Stepien and Baernstein 2006; Winseman et al. 2009; Wear and Zarconi 2008; Ahrweiler et al. 2014). Learning climate may be useful indirectly by positively contributing to these targeted educational interventions. Overall, research should continue to focus on identifying factors other than the learning climate that can maintain or improve residents’ empathy levels (Hojat et al. 2002b), especially given the noted decline in empathy among residents (Neumann et al. 2011). Studies comparing results from different specialties might provide valuable information on residents’ empathy.

### Strengths and limitations

This study adds to the existing knowledge on residents’ well-being and provides evidence on the possible role of residents’ learning climate in their work-related well-being. Its importance is underpinned by the international health care accreditation standards that require hospitals to manage matters of physician well-being (The Joint Commission 2009). In this multicenter study, we evaluated not only the association of the overall learning climate but also that of the specific learning climate domains with residents’ engagement, work satisfaction and empathy.

One limitation of this study is its cross-sectional design. We can only speculate about causal and non-causal influences armed with theory and existing best evidence and literature. Additionally, it is possible that there is uncontrolled confounding due to variables such as sleep deprivation and workload that could influence residents’ work-related well-being and learning climate (Helmich et al. 2015; Arah 2017). A potential bias in our data collection could be due to the order of filling out the measures, the timing of the survey, and the (well-being) state and willingness of the participants to respond on a web-based survey. As this study is based on data from two specialties, namely surgery and gynaecology, in the Netherlands, we caution against extrapolating our findings beyond these settings, and encourage further investigation in other settings.

### Implications

This and earlier studies strongly suggest that the learning climate positively influences residents work-related well-being (Bakker et al. 2007; Xanthopoulou et al. 2009; van Vendeloo et al. 2014; Llera and Durante 2014; Bakker et al. 2005). However, there is no consensus on an appropriate overall well-being scale at this moment. It would be interesting for future research to investigate the impact of learning climate on the overall well-being of residents. Lombarts et al. (2014) also found that the learning climate could positively influence individual faculty’s teaching performance. This justifies program directors’ focus on residents’ learning climate in striving for high-quality residency training. Clearly, validly and reliably measuring the learning climate, as we did by using the D-RECT tool, is only the first step in continuous improvement. From experience in our academic medical centers and other teaching hospitals, discussing these results among residents and faculty will help in exploring, committing to and implementing actual improvements. Hospital-wide committees for residency training, bringing together program directors as the responsible faculty for high-quality training, could further facilitate and disseminate best-practices in creating positive learning climates. Future research on other ways to improve the learning climate is recommended and may be instrumental for improving residency training programs, residents’ well-being and subsequently the quality of patient care delivery.

## Conclusion

A positive, stimulating and supportive learning climate is important in residency training. Climate seems to be positively related to work engagement and job satisfaction of residents. If these findings can be replicated and shown to be causal, they may suggest that work engagement and job satisfaction could be increased by improving the learning climate of residency training programs.

## Appendix

See Table 5.

**Table 5:** Items of the global job satisfaction and global specialty satisfaction measures

Global job satisfaction	I find my present clinical work personally rewarding
	Overall, I am pleased with my work
	Overall, I am satisfied with my current practice
	My current work situation is a major source of frustration
	My work in this practice has not met my expectations
Global specialty satisfaction	My specialty no longer has the appeal to me it used to have
	If I were to start my career over again, I would choose my current specialty
	I would recommend my specialty to a student seeking advice
